# Widespread Mutations in Voltage-Gated Sodium Channel Gene of *Cimex lectularius* (Hemiptera: Cimicidae) Populations in Paris

**DOI:** 10.3390/ijerph18020407

**Published:** 2021-01-06

**Authors:** Mohammad Akhoundi, Dahlia Chebbah, Denis Sereno, Anthony Marteau, Julie Jan, Christiane Bruel, Nohal Elissa, Arezki Izri

**Affiliations:** 1 Parasitology-Mycology Department, Avicenne Hospital, AP-HP, Sorbonne Paris Nord University, 93009 Bobigny, France; dahlia.chebbah@gmail.com (D.C.); anthonymarteau@hotmail.fr (A.M.); arezki.izri@aphp.fr (A.I.); 2 Service Parisien de Santé Environnementale, Sous-Direction de la Santé (SPSE), Mairie de Paris, 75019 Paris, France; nohal.elissa@paris.fr; 3 MIVEGEC, Institut de Recherche pour le Développement, Montpellier University, 34032 Montpellier, France; denis.sereno@ird.fr; 4 InterTryp, Institut de Recherche pour le Développement, Montpellier University, 34032 Montpellier, France; 5 Agence Régionale de Santé (ARS) Île-de-France, 35, rue de la Gare, 75935 Paris CEDEX 19, France; julie.jan@ars.fr (J.J.); christiane.bruel@ars.fr (C.B.); 6 Unité des Virus Emergents (UVE: Aix Marseille Univ, IRD 190, INSERM 1207, IHU Méditerranée Infection), 13005 Marseille, France

**Keywords:** *Cimex lectularius*, insecticides, pyrethroids, voltage-gated sodium channel (VGSC), SNP

## Abstract

Bed bugs, *Cimex lectularius* and *C. hemipterus,* are common blood-sucking ectoparasites of humans with a large geographical distribution, worldwide. In France, little is known about the status of bed bugs’ infestation and their resistance to insecticides, particularly, pyrethroids. Here, we aimed to find mutations in the *kdr* gene, known to be involved in resistance to insecticides. We gathered bed bugs from various infested locations, including 17 private houses, 12 HLM building complex, 29 apartments, 2 EHPAD, and 2 immigrants’ residences. A total of 1211 bed bugs were collected and morphologically identified as *C. lectularius*. Two fragments of the *kdr* gene, encompassing codons V419L and L925I, were successfully amplified for 156 specimens. We recorded sense mutation in the first amplified fragment (*kdr1*) in 89 out of 156 (57%) samples, in which in 61 out of 89 (68.5%) sequences, a change of valine (V) into leucine (L) V419L was observed. Within the second fragment (*kdr2*), a homozygous mutation was recorded in 73 out of 156 (46.7%) specimens at the codon 925. At this position, 43 out of 73 (58.9%) specimens had a sense mutation leading to the replacement of leucine (L) by isoleucine (I). Among 162 mutant sequences analyzed (89 for the *kdr1* fragment and 73 for the *kdr2* one), we detected single point mutation in 26.6%, while 73.4% presented the mutation in both *kdr1* and *kdr2* fragments. All modifications recorded in bed bug populations of Paris are described to be involved in the knockdown resistance (*kdr*) against pyrethroids.

## 1. Introduction

Bed bugs, *Cimex lectularius* and *C. hemipterus,* are obligate blood-sucking insects belonging to the Cimicidae family, which feed on human blood [1,2]. Since their resurgence in the late 1990s, bed bug infestation of human habitats has drastically increased, leading to growing concerns [3,4,5]. This resurgence can be attributed at least in part to increased international travel and the development of insecticide resistance [6,7]. *C. lectularius* is a cosmopolitan species found commonly in temperate regions, while *C. hemipterus* is mainly present in tropical and subtropical areas [8].

Regarding the reports implying the bed bugs’ involvement in harboring over 45 pathogens [9] and the competence of these insects in pathogenic agent transmission in the laboratory setting [10,11], there is still no evidence certifying their vectorial role in the endemic areas [1,12]. The discovery and widespread use of organochlorine DDT (dichlorodiphenyltrichloroethane) as a powerful insecticide in 1939, has led to a drastic decline in bed bug infestations [13]. Nevertheless, insecticide resistance is currently reported, worldwide [14]. The first description of resistance to insecticides (DDT) was documented in 1947 on Hawaii Island [15,16]. Because of DDT’s detrimental effects on human health and the environment, its use was banned in most Western countries since the 1970s [17]. During the 1950s and 1960s, organochlorine insecticides were replaced by organophosphates and carbamates due to their more effectiveness [18,19]. Organophosphate insecticides, such as chlorpyrifos and diazinon, together with carbamates, like propoxur, were insecticides of choice to control bed bugs. At the end of the 1970s, pyrethroid insecticides such as permethrin, cypermethrin, deltamethrin, and resmethrin were developed and considered alternatives. Unlike organochlorines or organophosphates, they are odorless with lower residual stability [14]. With the resurgence of bed bug infestation worldwide in the 1990s, a new generation of insecticides is available [20]. The extensive application of such commonly used insecticides is suspected of favoring the emergence of insecticide resistance in *C. lectularius* and *C. hemipterus* populations, worldwide. This emergence challenges the control management programs [14]. Pyrethroids are still among the most widely used insecticides against bed bugs, particularly in Europe [17,21,22].

Point mutations in the voltage-gated sodium channel (VGSC) gene reduce the target-site sensitivity for pyrethroids and DDT, causing “knockdown resistance” (*kdr*) [23,24]. Three point-mutations are reported from *C. lectularius* (V419L, L925I, and I936F) and currently nine from *C. hemipterus* (L899V, M918I, D953G, Y/L995H, V1010L, I1011F, L1014F, V1016E, and L1017F/S) [25,26]. These mutations act by substituting the amino acid sequence of the VGSC protein that prevents the insecticide acting on the nervous system [27,28].

Despite the wide use of chemical insecticides in France, as an essential part of control management by private pest control practitioners (PCPs) and municipalities, little is known about the bed bugs’ resistance status towards pyrethroids. The first study on the resistance of bed bugs to pyrethroids was reported by Durand et al. [22] in two apartment complexes (HLM) of Saint-Ouen city in suburb of Paris. In vivo tests of insecticide susceptibility performed against bed bugs collected from suburbs of Paris concluded low susceptibility toward bendiocarb [4]. Using a molecular approach, we investigated the occurrence and frequency of mutations in the pyrethroid resistance in bed bug specimens, gathered from various Paris areas.

## 2. Materials and Methods

### 2.1. Bed Bug Collections

Bed bugs were collected within private houses, apartments, HLM building complex, EHPAD (nursing home for the elders), and immigrants’ residences in Paris and surrounding cities. All inhabitants were questioned. The information on the date and history of the infestation and the possible infestation route, the history of treatment, and the chemical application were recorded individually. According to infestation signs, observed during the visual inspection, an infestation scale ranging from 0 (no infestation) to 5 (high level of infestation) was used to categorize the infested locations (Table 1).

Bed bugs were sampled using a handheld vacuum cleaner (Dyson V7 trigger) or by entomological forceps. To avoid excessive mortality during bed bugs’ collection, they were placed into 5 mL sterile mini-glass vials with a piece of folded bound paper, simulating an artificial shelter. Live bed bugs were brought to the laboratory and identified under a stereomicroscope (Olympus SZ61, Japan). The identification of bed bugs was performed based on the identification keys, published by Usinger (1966) [8] and Walpole (1987) [29]. All specimens were individually labeled and kept at −20 °C, for further molecular analysis.

### 2.2. Molecular Genotyping of the kdr Gene

The extraction of DNA was carried out using Chelex 10% (Bio-Rad, California, USA) [30]. The DNA concentration was then quantified by Qubit (Thermo Scientific, Waltham, USA). The amplification of two fragments of 474 and 744 bp within the ORF of the voltage-sensitive sodium channel gene (kdr-like gene) was carried out by conventional PCR. The reaction was performed in 25 μL of reaction mixture containing 0.3 μmol/L of *kdr-1f* (fwd): (5′-AACCTGGATATACATGCCTTCAAGG-3′) and *kdr-1r* (rev): (5′-TGATGGAGATTTTGCCACTGATG-3′) for amplification of first fragment and *kdr-2f* (fwd): (5′-GGAATTGAAGCTGCCATGAAGTTG3-3′) and *kdr-2r* (rev): (5′-TGCCTATTCTGTCGAAAGCCTCAG-3′) for amplification of second fragment, 200 μmol/L dNTPs, buffer (50 mmol/L KCl, 10 mmol/L Tris-HCl, pH 8.3, and 1 mmol/L MgCl2), and 2.5 U of *Thermus aquaticus* DNA polymerase (AmpliTaq Gold; Applied Biosystems, Foster City, California, USA). The samples were incubated at 95 °C for 10 min for denaturation, followed by 40 cycles at 94 °C for 40 s, 52 °C (first fragment)/55 °C (second fragment) for 40 s, and 72 °C for 40 s. The final extension was at 72 °C for 10 min. Negative and positive controls were used for each batch of PCR. Amplicons were analyzed using electrophoresis on a 1.5% agarose gel containing ethidium bromide. All amplified fragments were subjected to bilateral DNA sequencing. The sequences were aligned against their wild-type homologous sequences (GenBank accession numbers, GU123927 and GU123928) using Basic Local Alignment Search Tool (BLAST) (www.ncbi.nlm.nih.gov/BLAST). The presence of single nucleotide polymorphism (SNP), V419K and L925I, in two amplified fragments of the *kdr* gene (*kdr1* and *kdr2*) was searched in both forward and reverse sequences using BioEdit v7.0.0 software [31].

### 2.3. Ethics Approval

This study was conducted in accordance with the Declaration of Helsinki, and the protocol was approved by the Ethics Committee of Avicenne Hospital, France (Project identification code: 95/99/AVC/ESA).

## 3. Results

**Figure 1:**
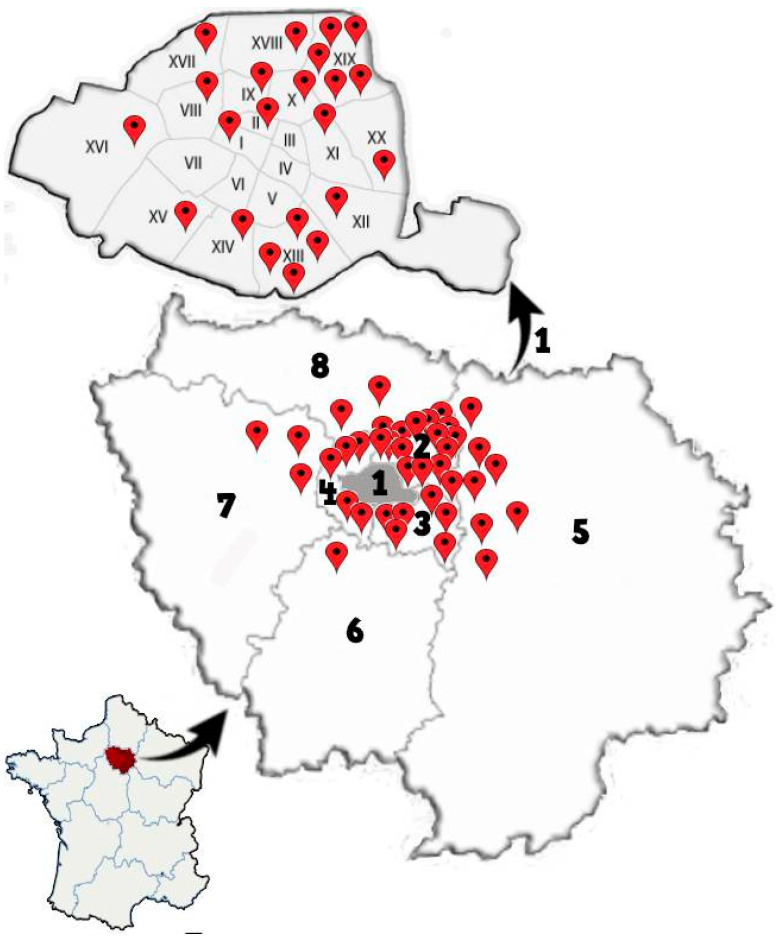
Geographical topology of the processed locations for bed bug sampling in Ile-de-France, France. 1. Paris arrondissements, 2: Seine-Saint-Denis, 3: Val-de-Marne, 4: Hauts-de-Seine, 5: Seine-et-Marne, 6: Essonne, 7: Yvelines, and 8: Val-d’Oise.

**Figure 2:**
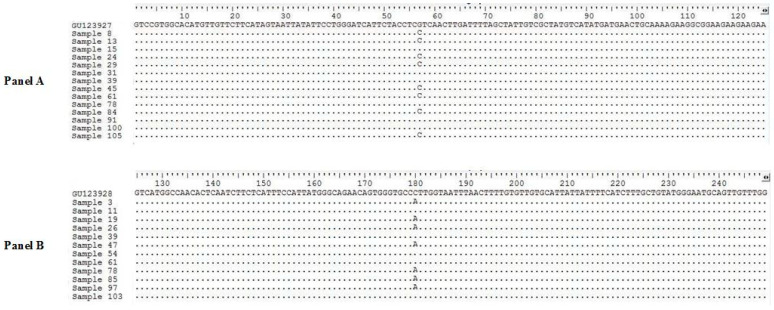
DNA sequence alignment of the voltage-gated sodium channel (VGSC) gene fragment encompassing nucleotides corresponding to the codons V419L (Panel A) and L925I (Panel B) in various *C. lectularius* specimens collected from Paris and suburb cities.

This study was carried out from January to June 2019, in collaboration with the public health department of Paris municipality, providing a preliminary list of potential infested locations in various geographic areas of Paris (see Figure 1). In total, 17 private houses, 29 apartments, 12 HLM building complex, 2 EHPAD, and 2 immigrants’ residences located in the 15 arrondissements of Paris (1,2,8,9,10,11,12,13,14,15,16,17,18,19 and 20) and 18 suburb cities (including Aubervilliers, Bobigny, Drancy, La Courneuve, Montreuil, Neuilly sur Marne, Pantin, and Stains (Seine-Saint-Denis department); Arcueil, Creteil, Nogent-sur-Marne, and Vincennes (Val-de-Marne department); Asnieres, Meudon, and Nanterre (Hauts-de-Seine department), Chilly Mazarin (Essonne department), Marly-le-Roi (Yvelines department), and Sarcelles (Val-d’Oise department)) were examined for the presence of bed bugs (Figure 1). Among them, 56 locations were infested by bed bugs, while in 6 locations, no bed bugs were noticed during inspection. Detail of bed bug specimens collected from different districts of Paris is given in Table 2. The number of insects collected varied, according to location ranging from one to more than 50 samples. Based on the scale of infestation level, most of the visited sites had the infestation of level 2 (22 locations) followed by level 3 (15 locations) (Table 1). A total of 1211 bed bugs belonging to various life stages (egg, nymph, adult male and female, unfed, and blood-fed) were collected. All adult specimens were morphologically identified as *C. lectularius.* The amplification of two fragments (encompassing codons 419 and 925) belonging to the VGSC gene was successfully carried out for 156 specimens, representing the geographical locations sampled. The alignment of the first amplified fragment (*kdr1*) with reference sequences collected in GenBank displayed the presence of a homozygous mutation at the codon 419 in 89 out of 156 (57%) specimens. Among these mutations, 61 out of 89 (68.5%) sequences revealed a change of valine (V) to leucine (L) V419L, while in the 28 (31.5%) remaining specimens, silent mutations were detected (Figure 2). Analysis of the second amplified fragment (*kdr2*) displayed the homozygous mutations at the codon 925 in 73 out of 156 (46.7%) specimens, in which 43 out of 73 (58.9%) specimens had a sense mutation, leading to the replacement of leucine (L) by isoleucine (I). In the remaining 30 (41.1%) specimens, mutations detected were silent (Figure 2). Among 162 mutant sequences analyzed (89 for the *kdr1* fragment and 73 for the *kdr2* one), a single mutation in one fragment was detected in 26.6% of cases. In contrast, 73.4% presented a single mutation in both amplified *kdr1* and *kdr2* fragments.

## 4. Discussion

With 64 million tourists in 2019, Paris belongs to the most visited city in the world. Therefore, bed bug infestations’ control remains a significant challenge in public health [32], with physical and psychological issues [33,34]. Despite increasing concerns reported by pest control practitioners (PCPs) and municipalities in the recent decade, no official report on the rate of bed bug infestation and control management success with chemical insecticides is available. Herein, we performed a survey of mutations occurring in the VGSC gene of *C. lectularius* populations collected from 15 out of the 20 “arrondissements” of Paris and 18 suburb cities. Our survey highlighted a high prevalence of bed bug infestation (56 out of 62 processed locations infested), with most infested sites being scale 2 (22/56, 39.2%). These findings are in accordance with two previous investigations carried out in the suburbs of Paris [4,22]. Furthermore, we did not find any correlation between the infestation levels defined in Table 1 and *kdr* point mutations in the processed bed bug populations. Based on epidemiological information gathered from inhabitants of the infested locations during inspections, 27.4% and 21.3% stated second-hand materials and infested objects (particularly travel suitcase), respectively, as the possible way of bed bug infestation, respectively, and 51.3% had no idea about the infestation source.

Since the introduction of synthetic insecticides, selection and adaptation of bed bugs might have occurred, allowing them to survive [35]. Among described mechanisms, target-site mutation and metabolic resistance are generally thought to be responsible for insecticide resistance in bed bugs [14]. The determination of bed bugs’ resistance status is usually performed by in vivo contact bioassays [4] or via identification of SNPs of target genes, known to be involved in metabolic resistance [22]. The VGSC expressed in the insect’s nervous system, is a target gene for which molecular markers of resistance to pyrethroids are described [20,36,37]. Bed bugs resistant to these insecticides display the point mutations in the VGSC gene. The presence of these SNPs correlates to the resistance in bed bugs against pyrethroids [20,36,37]. The L925I mutation in the *kdr* gene appears to be positively selected, more frequently than the V419L mutation, for pyrethroid resistance [36]. We reported a *kdr* gene haplotype with homozygous mutation of L925I and homozygous wild-type V419 codon, found in 61/89 (68.5%) and 43/73 (58.9%) of bed bugs collected in Paris and suburb cities. Strikingly a large majority (73.4%) of bed bugs collected in Paris bear both V419L and L925I mutations. To what extend these mutations impact the level of pyrethroid resistance needs further investigations. In particular, to firmly confirm bed bug resistance, further analysis using in vivo bioassays is required. These would shed light on the level of resistance to pyrethroid with single L925I or V419L mutations, compared to the combined effect of two mutations in specimens.

## 5. Conclusions

Our results highlight the predominance of pyrethroids resistance mutations in all populations collected. These would consequently affect these chemicals’ ineffectiveness in the control of bed bugs in Paris and suburb cities. These observations would prompt to reevaluate the intensive use of pyrethroids to control the bed bug infestations in Paris. The replacement of chemical treatments by nonchemical alternatives (such as dry heating or freezing) or the development of new eco-friendly alternative insecticides can reduce these insecticides’ harmful impacts.

## Figures and Tables

**Table 1:** Proposed infestation scale based on the signs observed by visual inspection.

Infestation Scale	Bed Bug Bite	Presence of Bed Bugs	Black Spot (Excretion)
0	No bite on residents	No bed bugs or different nymphal stages	No black spot or bug excrement on the bed, the mattress, and the area around the bed, on the bedding, and sheets
1	Bites on residents	Black spot or bed bug excretion on the bed	Presence of adults, eggs, and first or second nymphal stages
2	Bites on residents	Numerous black spots or bed bug excretion on the bed, mattress, and the area around the bed, on bedding and sheets, or the places the resident rests	Presence of adults, eggs, and various nymphal stages
3	Bites on residents	Numerous black spots or bed bug excretion on the bed, in other parts of the house	Presence of adults, eggs, and different nymphal stages, bed bugs are visible during the day
4	Bites on residents	Numerous black spots or bed bug excretion on the bed, in other parts of the house	Presence of different nymphal stages everywhere with high numbers, bed bugs are visible during the day
5	Bites on residents	Numerous black spots or bed bug excretion on the bed, in other parts of the house	Presence of different nymphal stages everywhere with high numbers, bed bugs are visible during the day, several bed bugs niches visible everywhere

**Table 2:** Details of bed bug infestations in various locations observed by visual inspection in Paris and suburb cities.

Location Type	Number of Inspected Locations		Number and Level of Infested Locations						Number of Collected Specimens
	Paris Arrondissements	Suburb Cities	0	1	2	3	4	5	
Private house	6	11	2	3	5	5	2	0	132
Apartment	11	18	3	5	9	7	3	2	195
HLM building complex	4	8	1	3	5	2	0	1	478
Migrant residence	0	2	0	0	1	1	0	0	389
EHPAD	1	1	0	0	2	0	0	0	17
Total	22	40	6	56					1211

EHPAD: nursing home for the elders.

## Data Availability

Data sharing not applicable.
